# Supplementary material for: Impacts of rapid mass vaccination against SARS-CoV2 in an early variant of concern hotspot
Source: Nat Commun. 2022 Feb 1;13:612. doi: 10.1038/s41467-022-28233-8 (PMC8807735; doi:10.1038/s41467-022-28233-8)
Supplement: Supplementary file 1 — Supplementary Information [file 41467_2022_28233_MOESM1_ESM.pdf]

Supplement

The effects of rapid mass vaccination against SARS-CoV-2 and its Variants-of-Concern: Evidence from an early VoC hotspot

Jörg Paetzold, Janine Kimpel, Katie Bates, Michael Hummer,
Florian Krammer, Dorothee von Laer and Hannes Winner

January 5, 2022

Contents

1 Data sources **2**

2 Institutional background on vaccination campaign **2**

3 Supplementary Tables **2**

4 Supplementary Figures **5**

5 References **16**

1 Supplementary Note 1. Data sources

For our retrospective observational study, we used data from the Austrian epidemiological reporting system (Österreichisches Epidemiologisches Meldesystem, EMS). These data are collected by the Austrian National Public Health Institute (Gesundheit Österreich GmbH, GÖG), along with information on hospital admissions due to COVID-19 diagnosis. Our database comprises municipality/district-level epidemiological data on all confirmed SARS-CoV-2 PCR-positive cases, sequencing (VoCs), and all hospital/ICU admission cases. Furthermore, confirmed cases can be disaggregated by four different age groups: 20–34, 35–49, 65–79 and above 80. Unfortunately, our data does not allow to disaggregate by age for those below the age of 20, which is an age group that was only partly offered a vaccine in the campaign (16 years) (see also Bathke, Happ and Hummer 2021).

Sequencing data is only available for the state of Tyrol, which responded with comprehensive sequencing of almost all SARS-CoV-2 PCR-positive cases after the large outbreaks of Beta and Alpha/E484K in February 2021. Vaccination data ("e-Impfpass") is only available for the state of Tyrol, since the federal states are responsible for the roll-out of the national vaccination plan. Our sample is based on the universe of all Austrian districts (all Tyrolean districts in the case of the vaccination and sequencing data) and all municipalities within those districts. We employed all infections, VoC cases (i.e., Beta, B1.1.7/E484K and Delta), hospital and ICU admissions recorded for those geographical units.

2 Supplementary Note 2. Institutional background on vaccination campaign

The vaccination campaign in Schwaz was limited to residents of the district of Schwaz. Every resident at the age of 16 or above who had his or her main legal residence in the district prior the announcement of the campaign (3rd of March 2021) was invited to get vaccinated. The vaccination campaign was advertised in local media such as newspapers and radio stations. The residence requirement was controlled and enforced at the vaccination centres where citizens received their doses. Only in the case of opened but unused jabs at the end of the campaign weekend, residents from towns outside of Schwaz were asked if they wanted to receive the vaccine. We do not have data to examine the exact extent of this practice, but anecdotal evidence as well as Figure 1 from the manuscript suggest that this effect was only negligible.

3 Supplementary Tables

Supplementary Table 1 summarizes the epidemic profiles of Schwaz and the control group used in the synthetic control group (SC) approach. It shows SARS-CoV-2 infection spread prior to the vaccination campaign, population size, geographical area size and the number of municipalities within a district. The series on cumulative infections end on the 11th of March and start three weeks (21 days) before the vaccination campaign on the 18th of February 2021 (i.e., "day 2" corresponds to the 19th of February and "day 21" to the 10th of March). Supplementary Table

2 shows pre-treatment characteristics of municipalities in Schwaz and the ones along the border of the neighbouring districts of Kufstein and Innsbruck-Land. The results of the corresponding (two-sided) t-test on the difference between the entries of the border municipalities and the ones in Schwaz are reported in the last column of the supplementary table.

Supplementary Table 1: Pre-treatment profiles for Schwaz and the synthetic control group

**Variable**	**Schwaz**	**Synthetic Schwaz**
Infections (day 2)	28.4	27.2
Infections (day 8)	184.7	189.7
Infections (day 14)	355.2	352.1
Infections (day 21)	465.3	470.6
Population	84,456	45,656.6
Area (km^2)	1,843.2	1,186.7
Municipalities within district	39	33.2
RMSPE		12.9

Notes: Infections represent cumulative numbers and are measured per 100,000 inhabitants. The chosen donors include Hartberg-Fürstenfeld (24.1%), Hermagor (10.6%), Liezen (0.5%), Reutte (63.8%) and Steyr Stadt (1.1%). The weights for the chosen districts of donor group are reported in parentheses. All other Austrian districts receive zero weight. The RMSPE (root mean squared prediction error) measures the difference in infections per 100,000 between Schwaz and the synthetic control group for all pre-treatment periods.

Supplementary Table 2: Pre-treatment profiles for municipalities in Schwaz and the ones along the neighbouring districts Kufstein and Innsbruck-Land

**Variable**	**Treatment Group**	**Control Group**	**p-value**
Number of municipalities	39	10	
Population	2137.1	3431.0	0.158
Population per km^2	148.9	252.2	0.199
Share females (%)	49.3	49.6	0.593
Share age $<$ 16 (%)	18.3	18.2	0.956
Share age 16–25 (%)	12.4	11.6	0.037
Share age 26-65 (%)	54.2	54.6	0.543
Share age $>$ 65 (%)	15.1	15.6	0.575
Migrants per capita	12.7	11.5	0.434
Share secondary education (%)	7.2	9.3	0.107
Share of commuters (%)	71.4	69.3	0.933
Unemployment rate (%)	5.8	5.4	0.334

Notes: Treatment and control group consists of municipalities in Schwaz and the municipalities along the border of the neighbouring districts and Kufstein and Innsbruck-Land. Columns (1) and (2) report mean values, column (3) the p-value of a two-sided t-test with unequal variances on the differences in the respective characteristics.

We do not have district-level daily vaccination data for districts outside of Tyrol prior the campaign,

which prevents us from including vaccination levels in the SC method in order to determine the choice of the control units. However, given that Supplementary Table 1 shows relatively small differences between pre-treatment infection rates in the treatment and the control group, we would expect only a small gain from including vaccination data in this process. In fact, Figure 1 of the manuscript shows that vaccination levels of Schwaz and all other Tyrolean districts (the only districts for which we have vaccination data) have been very similar before the campaign, which was likely the same case also for all other Austrian districts due to the uniform execution of the national vaccination plan.

Supplementary Table 3 shows the point estimates of the two-period difference-in-difference (DID) estimates, on which the percentage values of Table 1 in manuscript are based on.

Supplementary Table 3: Two-period DID

**DID-comparison**	**New infections**	**VoC**	**Hospitalization**	
			General admission	ICU
**Schwaz vs. border**	–101.49	–114.4	–6.29	–4.09
	[–150.0 ; –52.9]	[–181.0 ; –47.9]	[–10.5 ; –2.1]	[–7.0 ; –1.2]
No. of observations	1,323	1,323	3,619	3,619

Notes: Sum of weekly infections, variants of concern (VoC) and hospitalization in 100,000. VoC include the following mutants: Beta, Alpha/E484K, Delta. The two-period DID is based on a before-/after-comparison between Schwaz and its border municipalities. 95%-CI in brackets.

4 Supplementary Figures

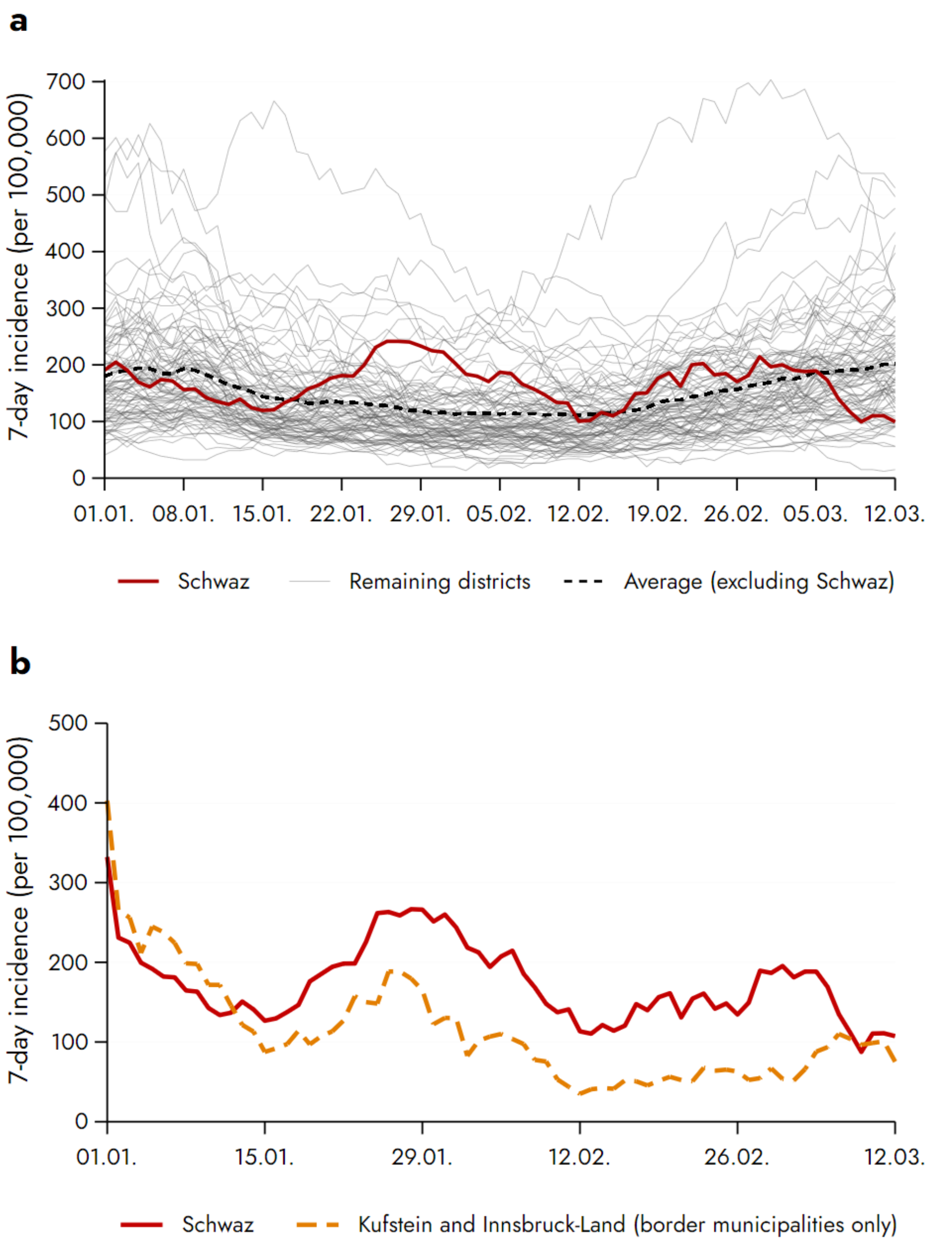

Supplementary Figure 1: 7-day incidence in treatment- and control group before the vaccination campaign

Notes: This supplementary figure shows the 7-day incidence (per 100,000) in the pre-treatment period starting on the 1st of January and ending on the 12th of March 2021 (one day after the first dose of the vaccination campaign in Schwaz was administered). Panel **a** shows the pre-trend for the Austrian districts. Schwaz is indicated by the red line and the remaining Austrian districts are represented by the solid grey lines. The dashed line represents the average 7-day incidence of all Austrian districts excluding Schwaz. Panel **b** shows the pre-trend for the municipalities along the border. Municipalities of Schwaz are in red, the ones of Kufstein are in orange and the ones of Innsbruck-Land are in blue. The observational (pre-treatment) period starts on the 1st of January 2021 and ends at the 12th of March 2021 (one day after the first dose of the vaccination campaign in Schwaz was administered).

Supplementary Figure 2 describes the results of a placebo test, where we applied the SC method sequentially on each of the 92 districts in the donor pool ("placebo units"), using the date of the roll-out of the first dose in Schwaz as the treatment date (11th of March). It shows the distribution of differences between the treated units and their respective synthetic control units for Schwaz (red line) and each of the 92 placebo units (grey lines) for daily infections per 100,000 as respective outcome variable. Visual inspection shows that the treatment effect in Schwaz was much higher than for any other placebo unit. To evaluate the significance of the observed differences between Schwaz and the synthetic control group, we ranked the treatment effects of all 93 districts plotted in Supplementary Figure 2 in ascending order starting with the highest (negative) effects and employed a Fisher permutation test as proposed by Abadie, Diamond and Hainmueller (2010), leading to a p-value of 0.005 (see also Abadie 2021: 403). Supplementary Figure 3 shows the same permutation exercise for general hospital admissions (panel a) and ICU admissions (panel b). Notice again that the results for ICU admissions are less reliable given the small number of admissions at the district level.

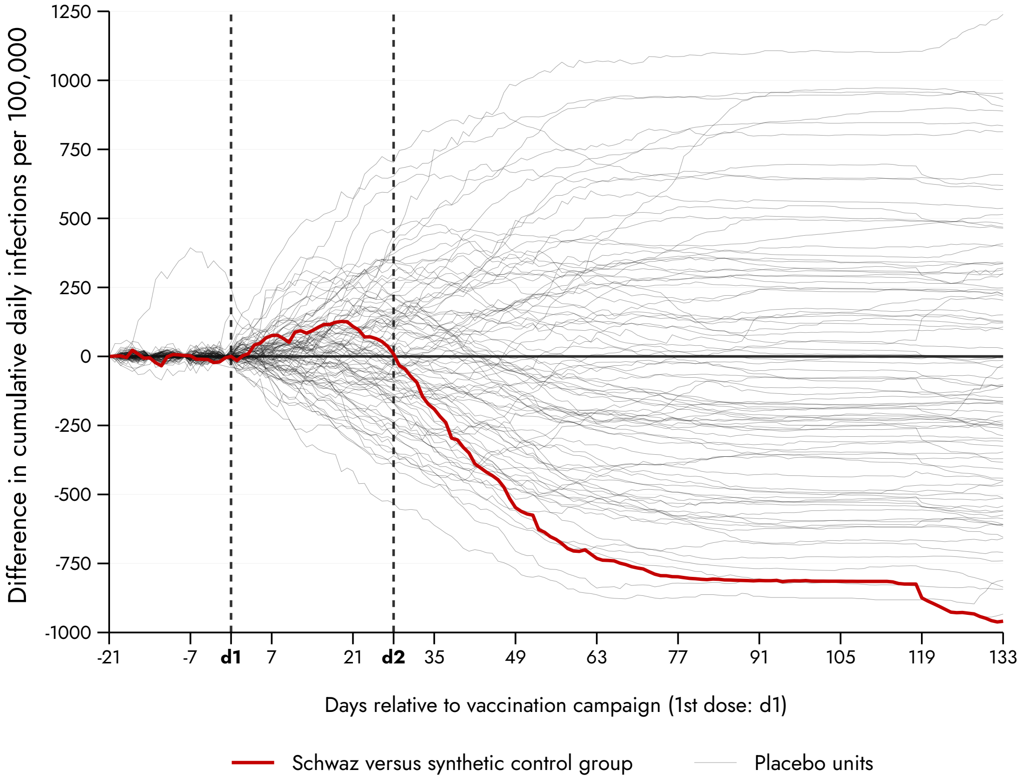

Supplementary Figure 2: Placebo-in-space for infections in Schwaz versus all donors

Notes: This supplementary figure describes a placebo test, where the SC method is sequentially applied on each district in the donor pool ("placebo units"), using the date of the first dose in Schwaz as the treatment date (11th of March). It shows the distribution of differences between the treated units and their respective synthetic control units for Schwaz (red line) and each of the 91 placebo units (grey lines). A positive (negative) difference indicates a higher (lower) transmission in the treated group relative to the synthetic control group. The horizontal axis shows the number of days relative to the vaccination campaign (dose 1, indicated by "$d1$"). The pre-treatment period started 21 days (three weeks) before the first dose, the post-treatment period ended 133 days (19 weeks) after the first dose. The vertical dashed lines represent the first dose ($d1$) and the second dose ($d2$) administered as part of the mass vaccination campaign.

a

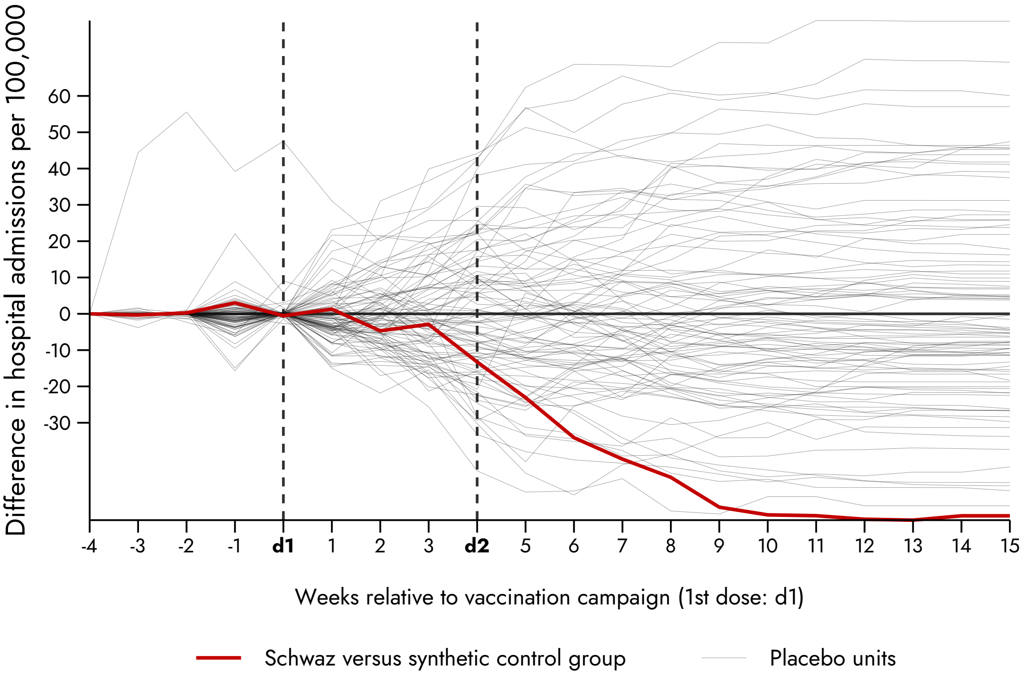

b

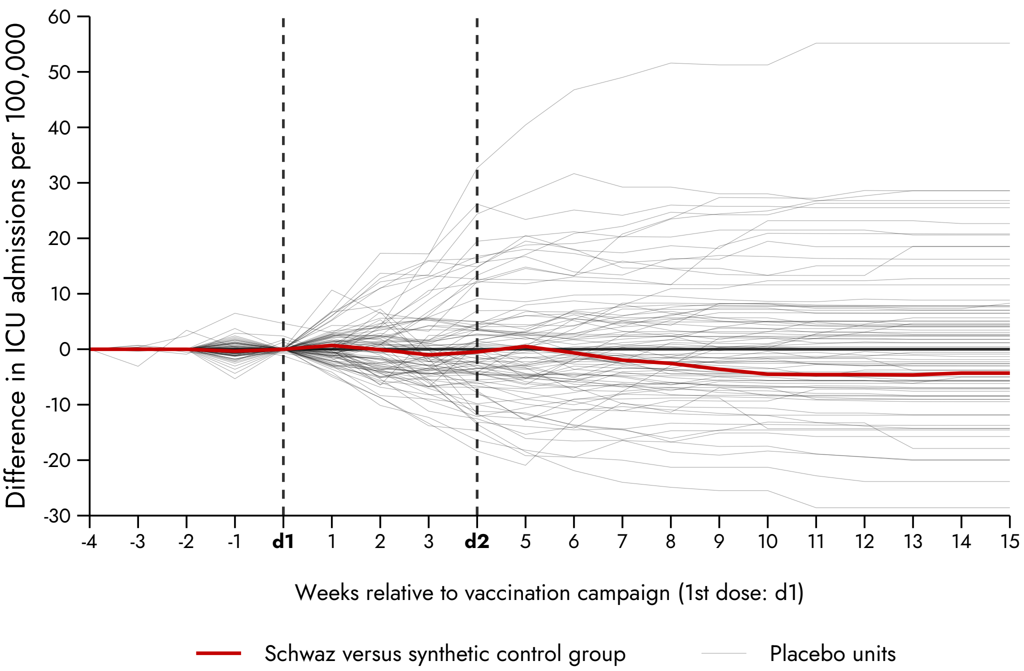

Supplementary Figure 3: Placebo-in-space for general and ICU admissions in Schwaz versus all donors

Notes: This supplementary figure describes the same placebo tests as in Supplementary Figure 2, but now relies on **a:** general hospital admissions, and **b:** ICU admissions. For further references see the notes in Supplementary Figure 2.

Supplementary Figure 4 provides an additional robustness test leaving out one of the chosen donors in the baseline control group (blue dashed line) one at a time. In some cases this increases and in others this lowers the difference in daily infections between the synthetic control group and Schwaz. In the paper, we reported 2,460 cumulative infections for the control group (and 1,510 ones for Schwaz) at the end of the observational period (i.e., 19 weeks after the first dose), decreasing to 2,160 infections in the ('worst') case leaving out Reutte which had a share of 63.8% in the baseline control group. However, this difference is still substantial, letting us conclude that our main result with regard to the effectiveness of the the vaccination campaign remains more or less unchanged.

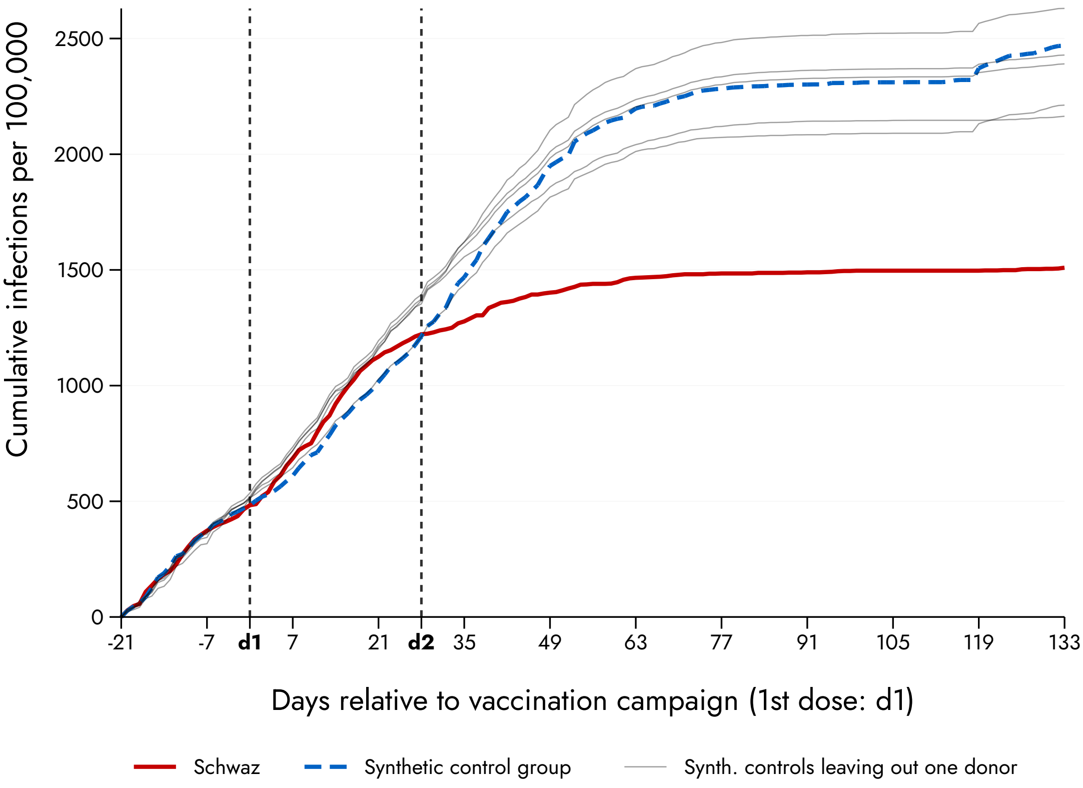

Supplementary Figure 4: Treatment effect of vaccination campaign in Schwaz with one-leave-out

Notes: This supplementary figure depicts cumulative daily infections (per 100,000) for Schwaz (solid red line) and the synthetic control group (dashed blue line). The blue dashed line corresponds to the baseline control group reported in the paper. The grey lines represent the results of a synthetic control group where one of the chosen donors in the baseline are left out one at a time. The horizontal axis indicates the number of days relative to vaccination campaign (dose 1). The pre-treatment period started 21 days (three weeks) before the first dose, the post-treatment period ended 133 days (19 weeks) after the first dose. The vertical dashed lines represent the first dose ($d1$) and the second dose ($d2$) administered in the vaccination campaign.

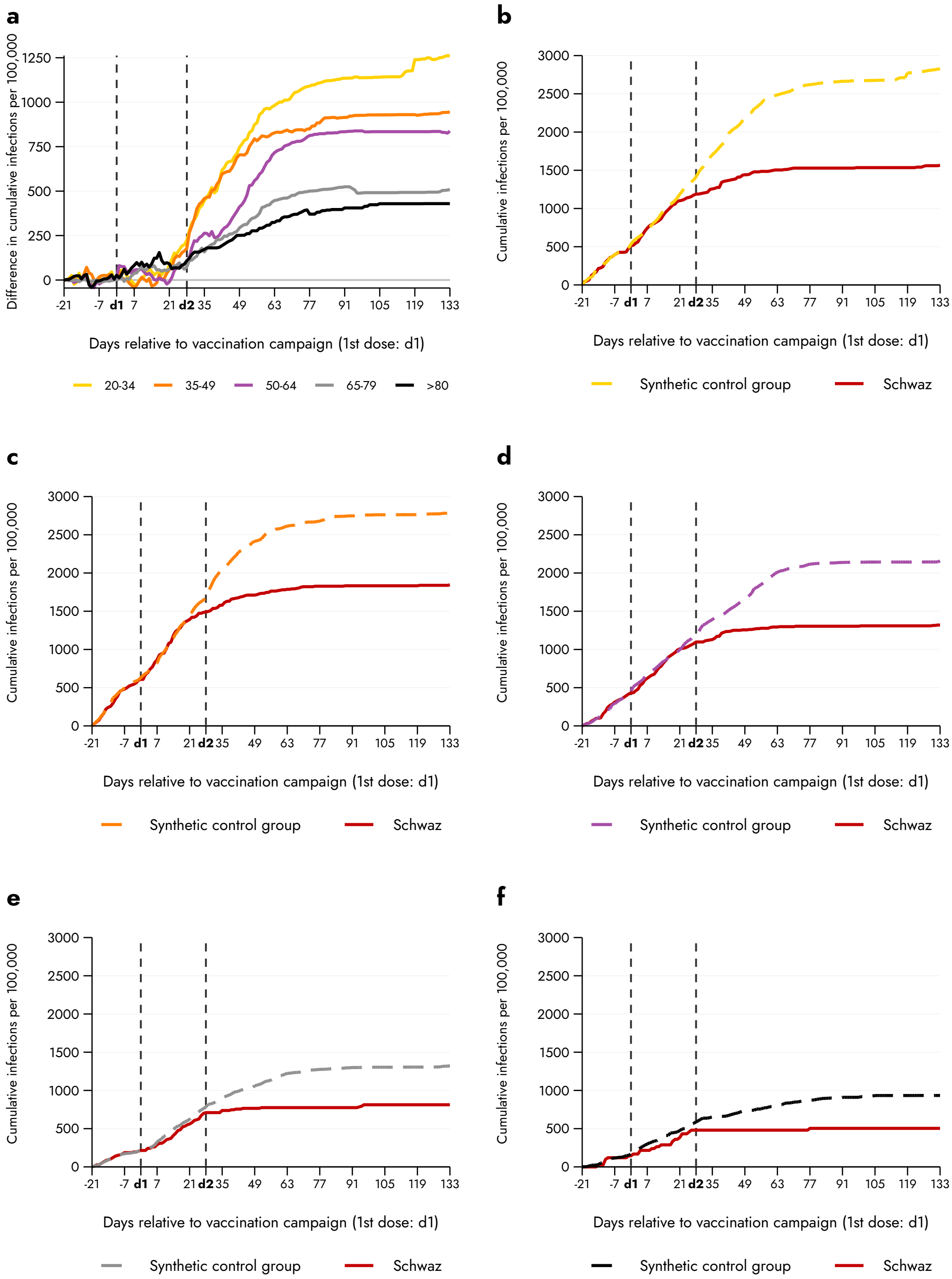

Supplementary Figure 5: Age-specific differences in cumulative daily infections between Schwaz and the synthetic control group

Notes: This supplementary figure plots cumulative daily infections (per 100,000) for each age cohort in the sample and compares the epidemic development in Schwaz (red solid line) with the ones in the synthetic control groups (dashed lines). The age cohorts are: **b:** 20–34 (yellow), **c:** 35–49 (orange), **d:** 50–64 (purple), **e:** 65–79 (grey), and **f:** >80 (black). For comparison, **a** depicts the absolute difference between Schwaz and the age-specific synthetic control groups. The pre-treatment period started 21 days (three weeks) before the first dose, the post-treatment period ended 133 days (19 weeks) after the first dose. The vertical dashed lines represent the first dose ($d1$) and the second dose ($d2$) administered as part of the mass vaccination campaign, respectively.

Supplementary Figure 6 plots the weekly incidence rates for hospitalizations and ICUs, corresponding to the cumulative measure shown in Figure 3 of the manuscript. Notice that the weekly incidence of ICU only includes low case numbers. For instance, since the start of the vaccination campaign there is only a total number of 11 ICU admission in Schwaz and 24.9 ICU admissions in the synthetic control group (the corresponding numbers for general admissions are 39 and 65.7).

a

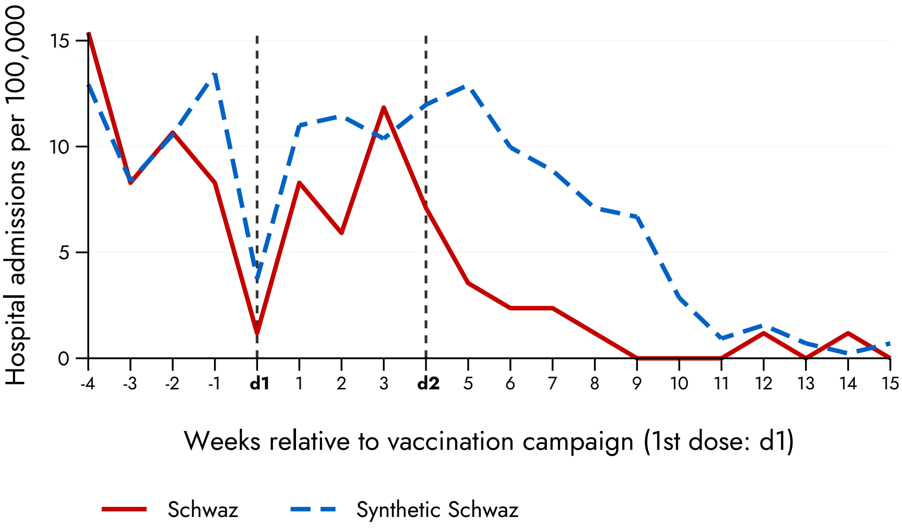

b

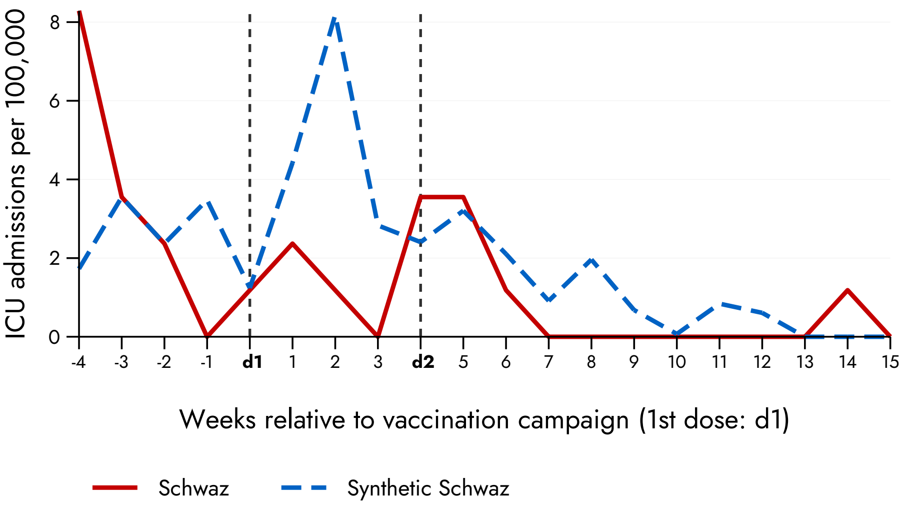

Supplementary Figure 6: Weekly hospital and ICU admissions in Schwaz versus synthetic control group

Notes: This supplementary figure shows the weekly hospital admissions (per 100,000) related to a confirmed SARS-CoV-2 infection for Schwaz and the synthetic control group. **a** relates to general hospital admissions, and **b** to ICU admissions. The horizontal axis shows the number of weeks relative to vaccination campaign (dose 1). The pre-treatment period started four weeks before the first dose, the post-treatment period ended 15 weeks after the first dose. The vertical dashed lines represent the first dose ($d1$) and the second dose ($d2$) administered as part of the mass vaccination campaign.

While almost all non-pharmaceutical interventions (such as school measures, or curfew restrictions) were identical for Schwaz and the different control groups, there was an additional SARS-CoV-2 test requirement between the 11th of March and the 8th of April when crossing the border of the district. This test requirement may have affected mobility as well as the spread of infections. However, we analysed Google mobility data and found, if anything, a slight increase in mobility for Schwaz relative to the synthetic control group Supplementary Figure 7.

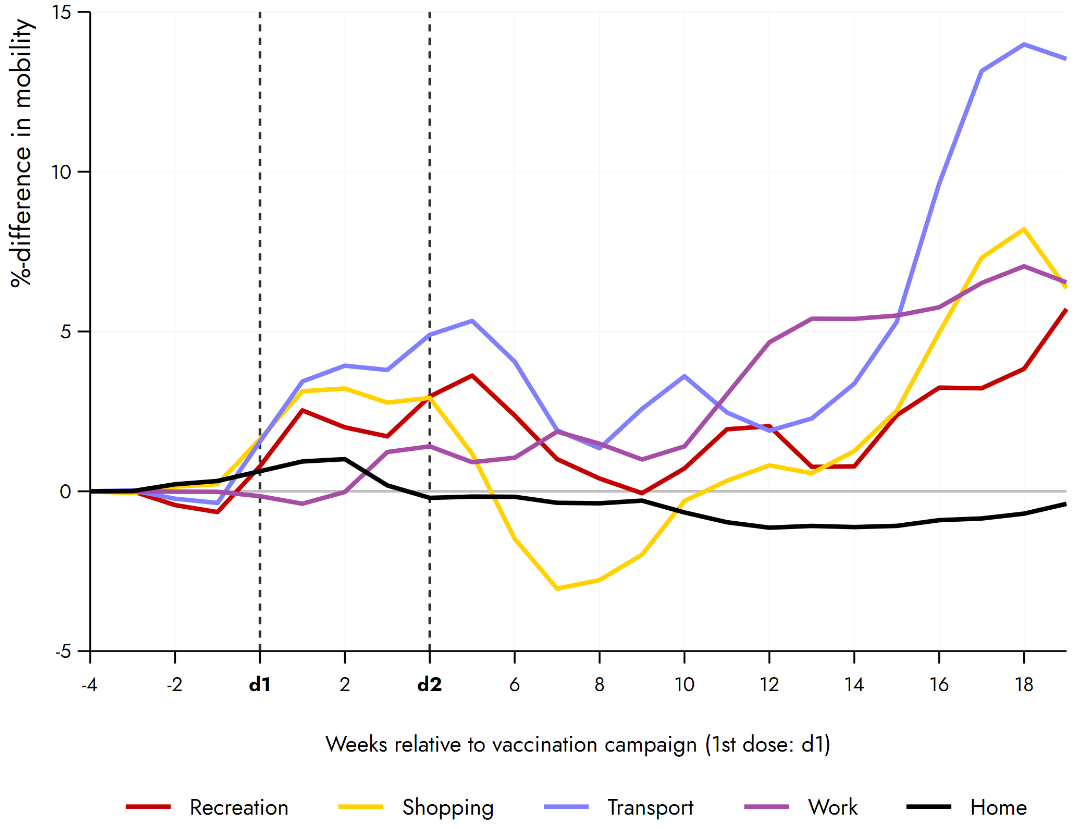

Supplementary Figure 7: Mobility patterns in Schwaz versus synthetic control group

Notes: This supplementary figure depicts the weekly difference in various mobility measures between Schwaz and the synthetic control group. The mobility measures are based on the Google COVID-19 Community Mobility Reports showing visits and length of stay for five different places and occasions: Recreation (e.g., restaurants, cafes, shopping centres, museums or libraries), shopping (e.g., grocery markets, food warehouses, farmers markets or pharmacies), transport (e.g., public transport hubs such as subway, bus and train stations.), workplaces and residence (home). A positive difference indicates higher mobility in Schwaz than for the control group. The horizontal axis shows the number of weeks relative to the vaccination campaign (dose 1). The pre-treatment period started four weeks before the first dose, the post-treatment period ended 19 weeks after the first dose. The vertical dashed lines represent the first dose ($d1$) and the second dose ($d2$) administered as part of the mass vaccination campaign, respectively. Source: Google LLC "Google COVID-19 Community Mobility Reports". https://www.google.com/COVID19/mobility/ [August 10, 2021].

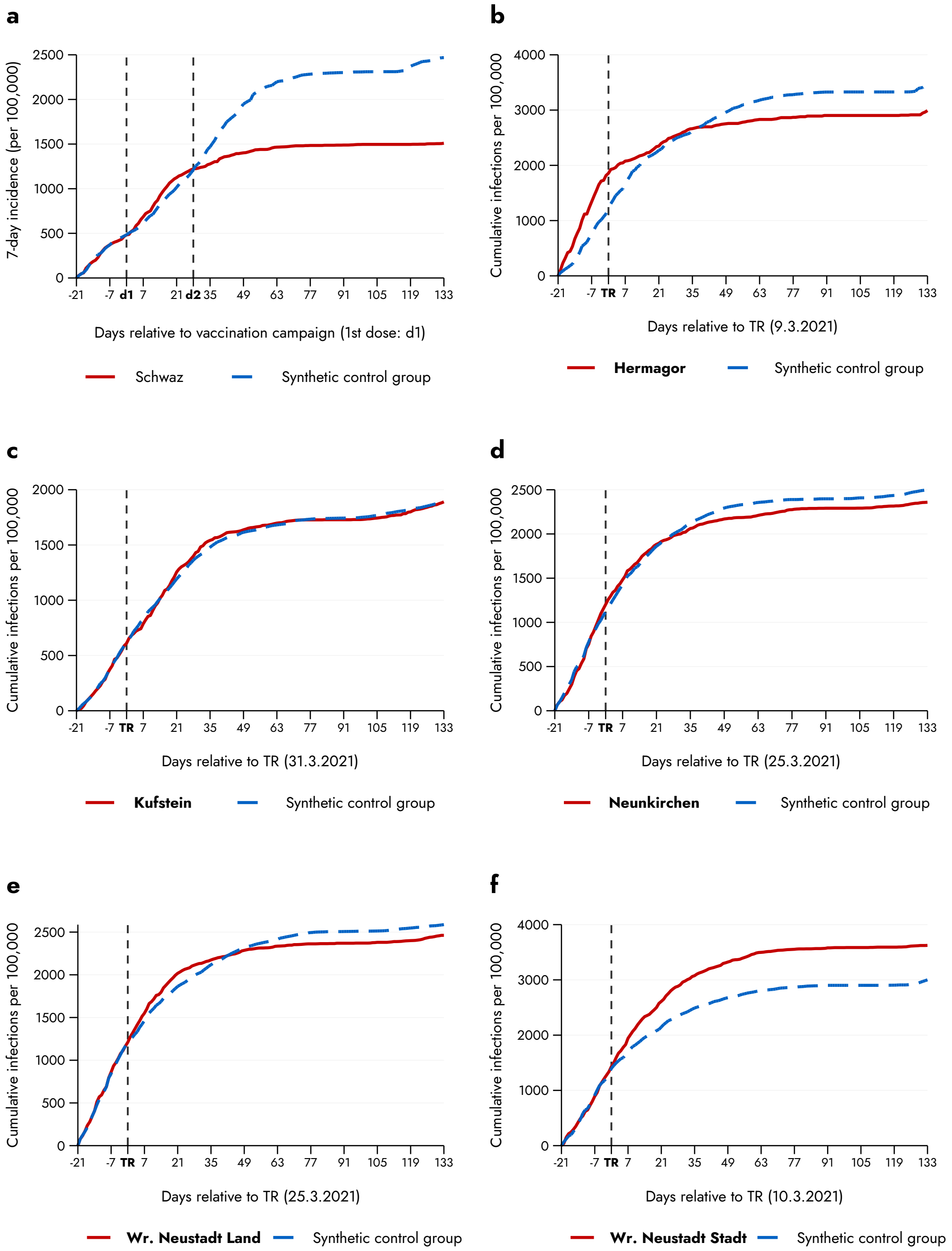

Supplementary Figure 8: Cumulative daily infections of Schwaz and NPI-districts versus synthetic control group

Notes: This supplementary figure depicts cumulative daily infections (per 100,000) for the five Austrian districts which had a test requirement (TR) in place for crossing district borders and the respective synthetic control group. These five districts are **b:** Hermagor, **c:** Kufstein, **d:** Neunkirchen, **e:** Wiener Neustadt Land, and **f:** Wiener Neustadt Stadt. For comparison, **a** depicts Schwaz versus its synthetic control group, which is identical to Figure 2 of the main text. The SC algorithm allows a selection of control units that reflect the pre-treatment trend very well. The only exception is Hermagor, where we observe considerable differences in outcomes between the treatment and the control unit in the pre-treatment period, which in turn makes the post-treatment comparison less reliable. The horizontal axis shows the number of days relative to the respective start of the test requirement (the corresponding starting dates are reported in the legend of the figures), which coincided with the first vaccination campaign week (dose 1) in Schwaz. The pre-treatment period started three weeks before the corresponding test requirement, the post-treatment period ended 19 weeks thereafter. The vertical dashed lines represent the first dose ($d1$) and the second dose ($d2$) administered as part of the mass vaccination campaign, respectively.

5 Supplementary References

1. Abadie A. Using synthetic controls: Feasibility, data requirements, and methodological aspects. Journal of Economic Literature; 59: 391-425 (2021).

2. Abadie A., A. Diamond and J. Hainmueller. Synthetic control methods for comparative case studies: Estimating the effect of California's tobacco control program. Journal of the American Statistical Association; 105: 493-505 (2010).

3. Bathke, A.C., M. Happ and M. Hummer. Indirekte Impfeffekte für Kinder und Jugendliche bei Durchimpfung der Erwachsenen. Vergleichende Fallstudie Schwaz/Tirol; Stand: 24.05.2021. Executive Policy Brief (unpublished manuscript), University of Salzburg (2021).
