# Supplementary material for: Impacts of rapid mass vaccination against SARS-CoV2 in an early variant of concern hotspot
Source: Nat Commun. 2022 Feb 1;13:612. doi: 10.1038/s41467-022-28233-8 (PMC8807735; doi:10.1038/s41467-022-28233-8)
Supplement: Supplementary file 3 — Reporting Summary [file 41467_2022_28233_MOESM3_ESM.pdf]

nature portfolio

Corresponding author(s): Joerg Paetzold

Last updated by author(s): Dec 18, 2021

Reporting Summary

Nature Portfolio wishes to improve the reproducibility of the work that we publish. This form provides structure for consistency and transparency in reporting. For further information on Nature Portfolio policies, see our Editorial Policies and the Editorial Policy Checklist.

Statistics

For all statistical analyses, confirm that the following items are present in the figure legend, table legend, main text, or Methods section.

n/a	Confirmed	
☐	☒	The exact sample size (*n*) for each experimental group/condition, given as a discrete number and unit of measurement
☐	☒	A statement on whether measurements were taken from distinct samples or whether the same sample was measured repeatedly
☐	☒	The statistical test(s) used AND whether they are one- or two-sided<br>*Only common tests should be described solely by name; describe more complex techniques in the Methods section.*
☐	☒	A description of all covariates tested
☐	☒	A description of any assumptions or corrections, such as tests of normality and adjustment for multiple comparisons
☐	☒	A full description of the statistical parameters including central tendency (e.g. means) or other basic estimates (e.g. regression coefficient) AND variation (e.g. standard deviation) or associated estimates of uncertainty (e.g. confidence intervals)
☐	☒	For null hypothesis testing, the test statistic (e.g. *F*, *t*, *r*) with confidence intervals, effect sizes, degrees of freedom and *P* value noted<br>*Give P values as exact values whenever suitable.*
☒	☐	For Bayesian analysis, information on the choice of priors and Markov chain Monte Carlo settings
☒	☐	For hierarchical and complex designs, identification of the appropriate level for tests and full reporting of outcomes
☒	☐	Estimates of effect sizes (e.g. Cohen's *d*, Pearson's *r*), indicating how they were calculated

Our web collection on statistics for biologists contains articles on many of the points above.

Software and code

Policy information about availability of computer code

Data collection	Standard epidemiological analyses were conducted using standard commands in STATA/MP 16.1 (ref. 36) as well as with the STATA package Synth (see http://fmwww.bc.edu/RePEc/bocode/s/synth.html). The STATA package Synth is based on the R package Synth, which is described in full detail in: Abadie A, Diamond A, Hainmueller J. Synth: An R Package for Synthetic Control Methods in Comparative Case Studies. Journal of Statistical Software;42(2013); doi: https://doi.org/10.18637/jss.v042.i13.<br>The codes to replicate all the statistical analysis are accessible using the following URL: https://github.com/hwin365/2021_schwaz.
Data analysis	Standard epidemiological analyses were conducted using standard commands in STATA/MP 16.1 (ref. 36) as well as with the STATA package Synth (see http://fmwww.bc.edu/RePEc/bocode/s/synth.html). The STATA package Synth is based on the R package Synth, which is described in full detail in: Abadie A, Diamond A, Hainmueller J. Synth: An R Package for Synthetic Control Methods in Comparative Case Studies. Journal of Statistical Software;42(2013); doi: https://doi.org/10.18637/jss.v042.i13.<br>The codes to replicate all the statistical analysis are accessible using the following URL: https://github.com/hwin365/2021_schwaz.

For manuscripts utilizing custom algorithms or software that are central to the research but not yet described in published literature, software must be made available to editors and reviewers. We strongly encourage code deposition in a community repository (e.g. GitHub). See the Nature Portfolio guidelines for submitting code & software for further information.

Data

Policy information about availability of data

All manuscripts must include a data availability statement. This statement should provide the following information, where applicable:

- Accession codes, unique identifiers, or web links for publicly available datasets
- A description of any restrictions on data availability
- For clinical datasets or third party data, please ensure that the statement adheres to our policy

For this study we used data from the Austrian epidemiological reporting system (Österreichisches Epidemiologisches Meldesystem, EMS). These data are collected by the Austrian National Public Health Institute (Gesundheit Österreich GmbH, GÖG), and is provided to the researchers through a restricted-access agreement. Access to this dataset can be be given to other researchers through direct application for data access to the GÖG. Sequencing and vaccination data is made available by the "Amt der Tiroler Landesregierung", which can be applied for via email (lwz@tirol.gv.at).

Field-specific reporting

Please select the one below that is the best fit for your research. If you are not sure, read the appropriate sections before making your selection.

☐ Life sciences ☒ Behavioural & social sciences ☐ Ecological, evolutionary & environmental sciences

For a reference copy of the document with all sections, see nature.com/documents/nr-reporting-summary-flat.pdf

Behavioural & social sciences study design

All studies must disclose on these points even when the disclosure is negative.

Study description	This is a retrospective observational quantitative study using regression analysis for causal inference.
Research sample	Our research sample comprises of municipality/district-level epidemiological data from the universe of all Austrian districts, and all municipalities within those districts. From this universe we selected (i) a control group of highly similar districts regarding previous infection spread, and (ii) adjacent municipalities along the border of Schwaz which were just excluded from the vaccination campaign. This dataset allows us to study the impact of this unique mass vaccination campaign. As outcome variables we employed the number of infections, VoC cases (i.e., Alpha, Beta, Delta), hospital and ICU admissions recorded for those geographical units.
Sampling strategy	No sampling as we used the universe of all Austrian districts.
Data collection	These data are collected by the Austrian National Public Health Institute (Gesundheit Österreich GmbH, GÖG). The source of this data is the Austrian epidemiological reporting system (Österreichisches Epidemiologisches Meldesystem, EMS), where all SARS-CoV2 cases must be recorded. The instruments used are entries into excel files, which are then converted to STATA dta files by GÖG. Researcher was not blinded to experimental condition or study hypothesis.
Timing	January 2021 - August 2021
Data exclusions	No exclusion
Non-participation	No dropout
Randomization	No randomization. Our study design is based on quasi-experimental variation in vaccine coverage between treatment and control regions. We show extensively in the paper as well as Supplementary that these regions are highly comparable regarding infection spread prior the vaccination campaign, as well as regarding many other characteristics/covariates.

Reporting for specific materials, systems and methods

We require information from authors about some types of materials, experimental systems and methods used in many studies. Here, indicate whether each material, system or method listed is relevant to your study. If you are not sure if a list item applies to your research, read the appropriate section before selecting a response.

Materials & experimental systems

n/a	Involved in the study
☒	☐ Antibodies
☒	☐ Eukaryotic cell lines
☒	☐ Palaeontology and archaeology
☒	☐ Animals and other organisms
☐	☒ Human research participants
☒	☐ Clinical data
☒	☐ Dual use research of concern

Methods

n/a	Involved in the study
☒	☐ ChIP-seq
☒	☐ Flow cytometry
☒	☐ MRI-based neuroimaging

Human research participants

Policy information about studies involving human research participants

Population characteristics	Characteristics of the population living in the border municipalities (=control group) and the district of Schwaz (treatment group) are described in the Supplementary Appendix Table 2.
Recruitment	We recruited the entire population with main residency in the treatment and control regions for our study. Self-selection should therefore be minimal.
Ethics oversight	The Ethics Committee of the University of Salzburg provided a declaration of no objection.

Note that full information on the approval of the study protocol must also be provided in the manuscript.
